# Beta Band Transcranial Alternating (tACS) and Direct Current Stimulation (tDCS) Applied After Initial Learning Facilitate Retrieval of a Motor Sequence

**DOI:** 10.3389/fnbeh.2016.00004

**Published:** 2016-01-22

**Authors:** Vanessa Krause, Anna Meier, Lars Dinkelbach, Bettina Pollok

**Affiliations:** Institute of Clinical Neuroscience and Medical Psychology, Medical Faculty, Heinrich-Heine-University DuesseldorfDuesseldorf, Germany

**Keywords:** alpha oscillations, beta oscillations, consolidation, motor control, motor learning, neuromodulation, primary motor cortex (M1), serial reaction time task (SRTT)

## Abstract

The primary motor cortex (M1) contributes to the acquisition and early consolidation of a motor sequence. Although the relevance of M1 excitability for motor learning has been supported, the significance of M1 oscillations remains an open issue. This study aims at investigating to what extent retrieval of a newly learned motor sequence can be differentially affected by motor-cortical transcranial alternating (tACS) and direct current stimulation (tDCS). Alpha (10 Hz), beta (20 Hz) or sham tACS was applied in 36 right-handers. Anodal or cathodal tDCS was applied in 30 right-handers. Participants learned an eight-digit serial reaction time task (SRTT; sequential vs. random) with the right hand. Stimulation was applied to the left M1 after SRTT acquisition at rest for 10 min. Reaction times were analyzed at baseline, end of acquisition, retrieval immediately after stimulation and reacquisition after eight further sequence repetitions. Reaction times during retrieval were significantly faster following 20 Hz tACS as compared to 10 Hz and sham tACS indicating a facilitation of early consolidation. tDCS yielded faster reaction times, too, independent of polarity. No significant differences between 20 Hz tACS and tDCS effects on retrieval were found suggesting that 20 Hz effects might be associated with altered motor-cortical excitability. Based on the behavioral modulation yielded by tACS and tDCS one might speculate that altered motor-cortical beta oscillations support early motor consolidation possibly associated with neuroplastic reorganization.

## Introduction

A vast amount of everyday activities relies on motor learning i.e., motor skill acquisition with fast performance improvements followed by motor consolidation resulting in long-term stabilization of the acquired skill (Reis et al., 2008). Motor stabilization is associated with less susceptibility to external interference and offline improvement without further practice (Robertson et al., 2004; Reis et al., 2008; Robertson, 2009). With memory consolidation motor control becomes automatic requiring less attentional demands. The primary motor cortex (M1) is relevant for motor sequence acquisition (Doyon and Benali, 2005; Hardwick et al., 2013) and consolidation (Robertson et al., 2005) as early as within 30 min after end of acquisition (Halsband and Lange, 2006; Sami et al., 2014).

A well-established paradigm to study motor sequence learning is the serial reaction time task (SRTT) comprising a sequence of button presses eliciting learning over time as indicated by faster reaction times (Nissen and Bullemer, 1987). SRTT acquisition and early consolidation can be facilitated by non-invasive transcranial direct and alternating current stimulation (tDCS/tACS; Nitsche et al., 2003c; Antal et al., 2008; Tecchio et al., 2010; Stagg et al., 2011). Both techniques offer the possibility to alter brain activity non-invasively by application of weak electrical currents. Anodal tDCS is likely associated with an increase of brain excitability by subthreshold neuronal membrane depolarization while cathodal tDCS likely yields decreased excitability by hyperpolarization (Nitsche et al., 2003a,b; Stagg and Nitsche, 2011). Anodal tDCS applied over M1 during learning of a SRTT facilitates sequence acquisition (Nitsche et al., 2003c; Stagg et al., 2011) and results in improved retrieval when applied immediately after learning (Tecchio et al., 2010). These studies suggest that tDCS is suitable to facilitate initial learning or early consolidation depending on stimulation timing.

tACS applies a sinusoidal waveform at specific frequencies alternating between anode and cathode. Although the exact underlying mechanisms are less well understood, evidence exists that tACS may interact with oscillatory brain activity by synchronization of oscillations with the applied external frequency (Helfrich et al., 2014). tACS at 10 Hz also facilitates learning of a SRTT but not at 1, 15, 30 and 45 Hz (Antal et al., 2008) – suggesting that tACS effects depend on the exact stimulation frequency. Neurophysiological after-effects have been shown up to 30 min after stimulation cessation (Herrmann et al., 2013) and may with prolonged stimulation duration be associated with altered excitability promoting synaptic plasticity by subthreshold modulation of membrane potentials (Antal and Paulus, 2012, 2013; Thut et al., 2011; Herrmann et al., 2013).

A previous magnetoencephalography (MEG) study showed changes of M1 oscillations during learning and early consolidation of a motor task at alpha (8–12 Hz) as well as beta (13–30 Hz) frequencies (Pollok et al., 2014). While changes of alpha oscillations may reflect reduced attentional demands after acquisition, altered beta oscillations might indicate functional reorganization associated with early consolidation (Orban et al., 2010). Moreover, the data provide evidence for the assumption that the magnitude of oscillatory beta suppression during learning is associated with superior consolidation. Although those data suggest a specific significance of M1 beta oscillations for motor sequence learning, their significance for early consolidation is not well understood yet.

The present study aims at investigating to what extent retrieval of a newly learned motor sequence can be differentially affected by tACS at 10 and 20 Hz. Assuming that tACS modulates oscillatory activity and that M1 oscillations at alpha and beta frequencies represent different functions, we hypothesized that tACS effects vary depending on stimulation frequency. Due to our previous MEG study (Pollok et al., 2014), we hypothesized an impact of tACS at beta frequency.

Since it has been hypothesized that increased motor-cortical excitability facilitates motor learning as well as consolidation, anodal and cathodal tDCS were contrasted in a second experiment in order to investigate whether modulation of M1 excitability yields effects comparable to those following tACS.

## Materials and Methods

### Participants

Participants were naïve with respect to the hypotheses and the exact study aims. By means of a between-subject design they were assigned to Experiment 1 – tACS or Experiment 2 – tDCS. Participants and the main investigator were blinded with respect to the exact stimulation parameters until completion of raw data acquisition. A second investigator was responsible for the application of the stimulation.

General exclusion criteria for study participation were history or family history of epileptic seizures, history of migraine, unexplained loss of consciousness, or brain related injury, history of other neurological or psychiatric disorders, pregnancy, intake of central nervous system-effective medication, cardiac or brain pacemaker. Most participants had never received transcranial electrical stimulation before. Written informed consent was given prior to participation. The study was accomplished with the approval of the local ethics committee and is in accordance with the Declaration of Helsinki. All participants were classified as right-handed by means of the Edinburgh Handedness Inventory (EHI; Oldfield, 1971) and had normal or corrected to normal sight. For right-handedness a minimum EHI score of 40 was required. *A priori* sample size calculation revealed a total sample size of 33 participants in order to realize effect sizes of 0.60 with a mixed design and a significance level of α < 0.05. In order to keep the number of participants constant across groups, 36 volunteers (12 per group) were recruited for Experiment 1. In Experiment 2, a total of 30 volunteers (15 per group) were included yielding slightly higher effect sizes of 0.65.

#### Experiment 1 – tACS

Experiment 1 comprised three stimulation groups receiving either 10, 20 Hz or sham tACS. Thirty-six volunteers were randomly assigned to the three groups. Participants receiving 10 Hz tACS (7 male, 5 female; EHI: 88.33 ± 5.62) were 26.17 (± 1.18) years on average, participants with 20 Hz tACS (8 male, 4 female; EHI: 98.33 ± 1.67) were 26.42 (± 1.18), and the sham group (7 male, 5 female; EHI: 92.50 ± 3.05) was 25.33 (± 0.94) years on average. Age and handedness did not differ significantly between groups (age: *F*_(2,35)_ = 0.26, *p* = 0.77; EHI: *F*_(2,35)_ = 1.74, *p* = 0.19).

#### Experiment 2 – tDCS

In Experiment 2, effects of anodal and cathodal tDCS were contrasted. A sham stimulation group was not included. Thirty volunteers were randomly assigned to either anodal or cathodal tDCS. The anodal tDCS group (7 male, 8 female; EHI: 90.00 ± 2.93) was 23.60 (± 1.29) years on average, participants receiving cathodal tDCS (7 male, 8 female; EHI: 92.00 ± 2.43) were 22.73 (± 0.78) years on average. Age and handedness did not differ significantly (age: *F*_(1,29)_ = 0.33, *p* = 0.57; EHI: *F*_(1,29)_ = 0.28, *p* = 0.60).

### Apparatus and Materials

#### Design

**Figure 1:**
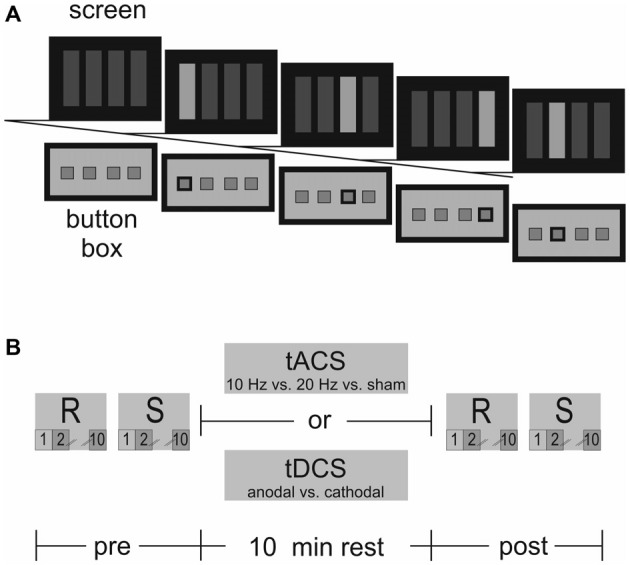
**(A)** Serial reaction time task (SRTT)–Presentation of the eight-digit SRTT in four target locations on the screen requiring participants to react with the first four fingers of the right hand on the response button box. From left to right: blank, thumb, middle, ring, index finger. **(B)** The SRTT was divided into identical trials prior to and immediately after 10 min tACS/tDCS at rest. Each trial was subdivided into a random (R) and sequential (S) condition. Selected sequences for data analysis are highlighted: baseline (T1; pre S_2_, R_2_), end of acquisition (T2; pre S_10_, R_10_), retrieval (T3; post S_2_, R_2_), reacquisition (T4; post S_10_, R_10_).

The eight-digit SRTT involved four-choice-reaction-time trials of stimuli presented in four target locations either at a sequential or random order (Figure 1A). Participants were kept naïve with respect to the sequential pattern within the SRTT and were introduced to the task as a measure of simple reaction times. The goal was to react as fast and as accurately as possible by pressing the corresponding button when a target on the screen changed its color from dark to light blue. The order of sequential and random SRTT trials was counterbalanced across participants and sessions. Within one session the order of trials was maintained. After initial SRTT acquisition participants were stimulated for 10 min at rest (Figure 1B). Immediately after stimulation, SRTT retrieval and reacquisition were tested. Reaction times were measured as dependent variable. The random condition served to distinguish between stimulation effects on motor sequence acquisition and on reaction times in general.

#### Serial Reaction Time Task (SRTT)

The SRTT is a well-established experimental paradigm eliciting motor sequence learning over time (Nissen and Bullemer, 1987). We applied an eight-digit SRTT requiring participants to react with the first four fingers (thumb (1), index (2), middle (3), ring finger (4)) of the right hand using a response button box fitted to the right hand that was connected to a standard Windows PC. Timing of the SRTT as well as recording of reaction times was realized by E-Prime^®^ (Psychology Software Tools Inc.). Stimuli were projected on a screen in front of the participants. Four blue rectangles were arranged horizontally along the middle of the screen against a black background at a distance of 2.66 m and with a visual angle of 12.87°. Stimuli remained present until the correct button was pressed. The next stimulus appeared after a 1 s delay. The sequence was 1–3–4–2–3–2–3–4 repeated 10 times in the sequential condition. During the random condition, fingers 1–4 were to press with the same number as in the sequential condition but in a random order.

#### Primary Motor Cortex (M1) – Stimulation Target Localization

Left M1 was localized by single transcranial magnetic stimulation (TMS) pulses in each participant. We used a standard figure of eight coil (MC-B70) connected to a MagPro stimulator (Medtronic, Minneapolis, MN, USA) placed tangentially to the scalp with the handle pointing backwards and laterally at about 45° away from the midline inducing an initial posterior-anterior current flow in the brain. The optimal cortical representation of the right first dorsal interosseous (FDI) muscle was localized by inducing motor-evoked potentials (MEP). By moving the coil in 0.5 cm steps anterior, posterior, medial, and lateral to this area, the exact localization of the spot which elicited the maximal FDI motor response was identified as stimulation target.

#### Transcranial Alternating and Direct Current Stimulation

Stimulation was applied for 10 min at rest by two 7 cm × 5 cm saline-soaked sponge electrodes on the skin surface (DC-Stimulator, Eldith, NeuroConn, Ilmenau, Germany). Electrodes were placed above the left M1 and the right orbita, respectively. Stimulation intensity was 1 mA (tDCS: single mode; tACS: sine mode) corresponding to 0.0286 mA/cm^2^ current density under the electrode. Impedance was kept below 5 kOhm. Stimulation parameters were in accordance with current safety guidelines of transcranial brain stimulation (Nitsche et al., 2003b; Rossi et al., 2009). Sham stimulation was realized by counterbalanced application with either 10 or 20 Hz in half of participants, respectively. The stimulator switched off automatically after 30 s. A masking flicker stimulus was presented for 10 min on the screen in front of the participants, since tACS at frequencies below 40 Hz is likely accompanied by visual flicker sensation (Paulus, 2010; Turi et al., 2013). During tDCS a blank gray screen was displayed. Participants were told to relax for 10 min, keeping their eyes open, and not talking.

Since stimulation was accomplished with a double-blind design, participants were to rate at the session’s end the type of stimulation. Furthermore, participants indicated their subjective rating confidence on a numerical rating scale from 1 (totally uncertain) to 10 (totally certain). Active stimulation was correctly identified by 24/54 participants (mean confidence 5.29 ± 1.30), sham stimulation by 10/12 participants (5.60 ± 0.85).

### Data Analysis

Reaction times i.e., onsets of button presses were logged in EPrime^®^ data files. The reaction time per sequence was measured as average of eight consecutive button presses during the sequential and random condition. The first sequence was discarded from further analysis to allow familiarization with the button box. We subdivided the motor learning process into four stages (baseline (T1), end of acquisition (T2), retrieval (T3), reacquisition (T4)). In both the sequential and random condition, we selected the second sequence as index for baseline and retrieval. The tenth sequence served as index for end of acquisition and reacquisition, respectively. Values outside confidence intervals (mean ± 2 standard deviations (SDs)) of individual and group data were discarded corresponding to 4.83% outliers in individual and 4.58% in group data. Mean reaction times are summarized in Table 1. Separate ANOVAs for the sequential and random condition were performed with within-subject factor *time* (T1 vs. T2 vs. T3 vs. T4) and between-subject factor *tACS* (10 Hz vs. 20 Hz vs. sham) for Experiment 1 and between-subject factor *tDCS* (anodal vs. cathodal) for Experiment 2. Greenhouse-Geisser corrected *p*-values are provided when appropriate. *P*-values were corrected for multiple testing with the sequential Bonferroni procedure (Holm, 1979). All statistical comparisons were calculated with IBM SPSS Statistics 22.

**Table 1:** **Group mean reaction times in ms (±SEM) for the sequential and random conditions during baseline (T1), end of acquisition (T2), retrieval (T3), and reacquisition (T4)**.

	SEQUENTIAL				RANDOM			
	T1	T2	T3	T4	T1	T2	T3	T4
10 Hz	571.14 (32.70)	486.97 (33.71)	453.23 (17.69)	413.67 (32.96)	589.28 (55.00)	592.95 (50.63)	502.23 (26.72)	453.01 (21.70)
20 Hz	503.29 (37.66)	447.77 (36.14)	365.21 (28.58)	363.29 (33.74)	557.88 (32.64)	492.75 (32.82)	449.76 (17.27)	469.41 (20.23)
Sham	487.78 (26.18)	463.12 (25.19)	463.58 (36.06)	320.39 (40.26)	568.88 (42.89)	523.85 (26.80)	474.02 (15.90)	479.36 (17.80)
Anodal	598.16 (39.26)	524.34 (20.69)	467.84 (18.88)	418.90 (28.03)	536.30 (17.56)	506.11 (16.05)	484.58 (14.52)	500.49 (14.91)
Cathodal	567.55 (23.79)	539.15 (30.29)	462.83 (23.23)	460.03 (26.19)	559.73 (28.29)	561.69 (29.14)	491.78 (18.35)	492.98 (11.26)

## Results

### Experiment 1 – tACS

ANOVA for the sequential condition revealed a significant main effect of *time* (*F*_(3,90)_ = 33.26, *p* < 0.001) and a significant *time × tACS* interaction (*F*_(6,90)_ = 2.68, *p* = 0.03). The main effect of *tACS* was not significant (*F*_(2,30)_ = 1.71, *p* = 0.20). In order to elucidate the significant *time × tACS* interaction, data were subsequently analyzed stepwise with respect to the following questions.

#### Does Initial Motor Learning Occur During the SRTT?

**Figure 2:**
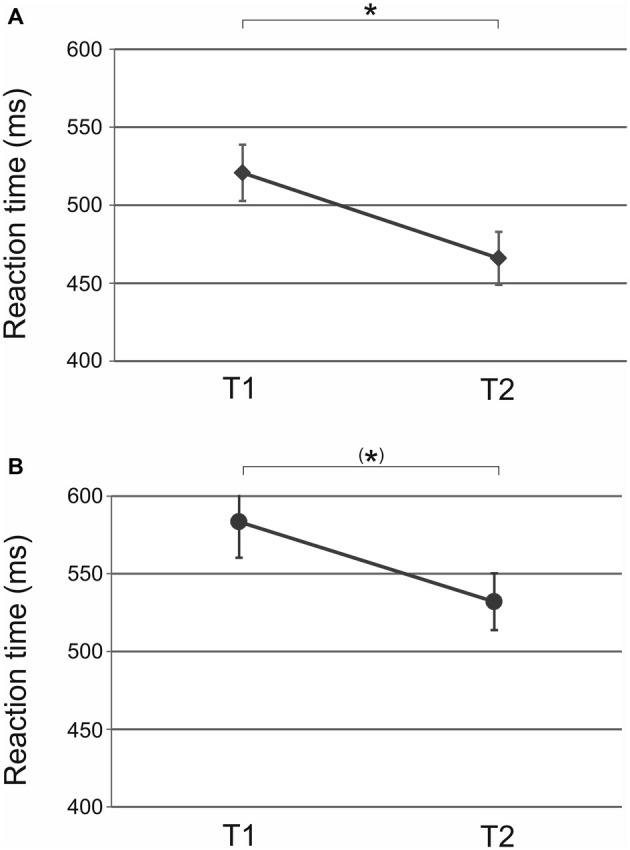
**Sequential condition: significant main effect of *time* from baseline (T1) to end of acquisition (T2) prior to (A) tACS und (B) tDCS.** Participants showed initial motor learning before stimulation was applied. Shown are mean values. Error bars indicate the standard error of the mean *(SEM)*. Asterisks indicate *p* < 0.05.

ANOVA with factors *time* (T1 vs. T2) and *tACS* (10 Hz vs. 20 Hz vs. sham) revealed a significant main effect of *time* (*F*_(1,31)_ = 15.50, *p* < 0.001; Figure 2A) but not *tACS* (*F*_(2,31)_ = 1.08, *p* = 0.35) or *time × tACS* interaction (*F*_(2,31)_ = 1.55, *p* = 0.23). Reaction times speeded up from baseline (T1) to end of acquisition (T2) indicating that participants showed motor learning over the course of ten repetitions prior to stimulation (*t*_(33)_ = 3.81, *p* < 0.01). This result indicates that prior to stimulation the performance did not differ between groups.

#### Does tACS Differentially Affect SRTT Retrieval?

**Figure 3:**
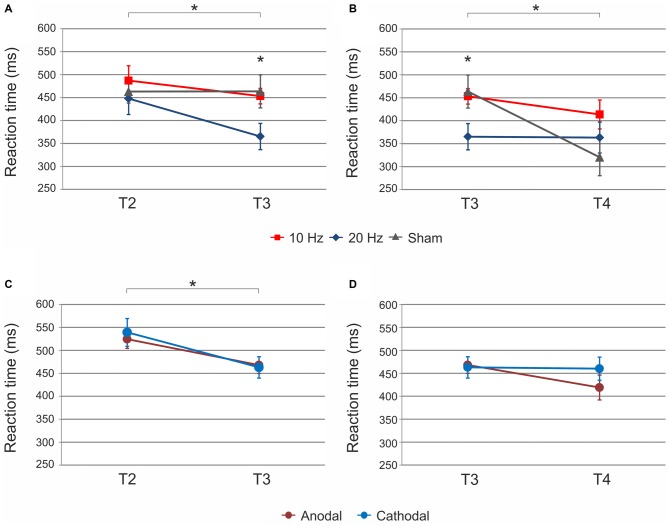
**Sequential condition: (A)** Significant main effect of *time* and *time* × *tACS* interaction from end of acquisition (T2) to retrieval (T3) immediately after tACS. Retrieval after 20 Hz tACS was characterized by significantly faster reaction times as compared to 10 Hz and sham tACS. **(B)** Significant main effect of *time* and *time × tACS* interaction from retrieval (T3) to reacquisition (T4). Reaction times significantly speeded up following sham tACS. Reaction times did not differ between stimulation types after reacquisition. **(C)** Significant main effect of *time* following tDCS. Reaction times significantly speeded up independent of polarity. **(D)** No significant effect of tDCS on reacquisition was found. Shown are mean values. Error bars indicate the SEM. Asterisks indicate *p* < 0.05.

ANOVA with factors *time* (T2 vs. T3) and *tACS* (10 Hz vs. 20 Hz vs. sham) revealed a significant main effect of *time* (*F*_(1,30)_ = 9.87, *p* < 0.01) and a significant *time × tACS* interaction (*F*_(2,30)_ = 3.27, *p* = 0.05; Figure 3A). The main effect of *tACS* was not significant (*F*_(2,30)_ = 2.37, *p* = 0.11).

In order to further elucidate the significant *time × tACS* interaction, we performed *post hoc* *t*-tests for independent samples. While there was no significant difference between stimulation groups at the end of acquisition (T2) i.e., prior to tACS (10 Hz vs. 20 Hz *t*_(20)_ = 0.79, *p* = 0.44, 10 Hz vs. sham *t*_(21)_ = 0.57, *p* = 0.57, 20 Hz vs. sham *t*_(21)_ = −0.35, *p* = 0.73), reaction times significantly differed between stimulation groups during retrieval (T3) immediately after tACS cessation. In detail, 20 Hz tACS yielded significantly faster reaction times at T3 than 10 Hz (*t*_(21)_ = 2.56, *p* = 0.04) and sham (*t*_(22)_ = −2.14, *p* = 0.04). No significant difference was found between 10 Hz and sham tACS (10 Hz vs. sham *t*_(21)_ = −0.25, *p* = 0.81). Furthermore, *t*-tests for dependent samples were calculated in order to determine reaction time differences between T2 and T3 within each stimulation group. Reaction times significantly decreased following 20 Hz tACS (T2 vs. T3: *t*_(10)_ = 4.02, *p* = 0.01) and 10 Hz tACS (T2 vs. T3: *t*_(9)_ = 3.49, *p* = 0.01) but not sham (T2 vs. T3: *t*_(11)_ = −0.01, *p* = 0.99). The data suggest that the significant interaction was mainly driven by 20 Hz tACS yielding significantly faster reaction times than 10 Hz and sham tACS at T3.

In order to investigate the possibility whether the beneficial 20 Hz effect on reaction times during sequence retrieval occurred at the expense of accuracy, we analyzed error rates as percentage of incorrect button presses prior to stimulation as compared to after stimulation. In general, error rates were low: 10 Hz: 1.96 ± 0.44% pre and 2.33 ± 0.82% post stimulation, 20 Hz: 2.95 ± 0.55% pre and 3.21 ± 0.95% post stimulation, sham: 3.28 ± 0.51% pre and 2.33 ± 0.30% post stimulation. ANOVA with factors *time* (pre stimulation vs. post stimulation) and *tACS* (10 Hz vs. 20 Hz vs. sham) revealed neither significant main effects (*time* *F*_(1,30)_ = 0.07, *p* = 0.80; *tACS* *F*_(2,30)_ = 0.74, *p* = 0.49) nor interaction (*F*_(2,30)_ = 1.25, *p* = 0.30). This renders a significant impact of changes in error rates on changes in reaction times unlikely.

#### Does tACS Differentially Affect Subsequent SRTT Reacquisition?

ANOVA with factors *time* (T3 vs. T4) and *tACS* (10 Hz vs. 20 Hz vs. sham) revealed a significant main effect of *time* (*F*_(1,32)_ = 15.50, *p* < 0.001) and a significant *time × tACS* interaction (*F*_(2,32)_ = 7.49, *p* < 0.01; Figure 3B). The main effect of *tACS* was not significant (*F*_(2,32)_ = 1.34, *p* = 0.28).

The interaction was further elucidated by *t*-tests for independent samples and was primarily explained by significantly faster reaction times during retrieval (T3) following 20 Hz as compared to both other stimulation conditions (20 Hz vs. 10 Hz *t*_(21)_ = 2.56, *p* = 0.04; 20 Hz vs. sham *t*_(22)_ = −2.14, *p* = 0.04), while this difference between stimulation groups did not persist after sequence reacquisition (T4; 10 Hz vs. 20 Hz *t*_(21)_ = 1.07, *p* = 0.30, 10 Hz vs. sham *t*_(21)_ = 1.77, *p* = 0.09, 20 Hz vs. sham *t*_(22)_ = 0.82, *p* = 0.42). Additional *t*-tests for dependent samples to compare reaction time differences between T3 and T4 showed a significant reaction time improvement from retrieval to reacquisition after sham stimulation (T3 vs. T4: *t*_(11)_ = 4.47, *p* < 0.01). 20 Hz tACS (T3 vs. T4: *t*_(11)_ = 0.09, *p* = 0.93) and 10 Hz tACS (T3 vs. T4: *t*_(10)_ = 1.55, *p* = 0.30) yielded no significant reaction time improvement from T3 to T4.

In the last step, the influence of the order of trials on the observed effects was elucidated. The ANOVA with factors *time* (T1 vs. T2 vs. T3 vs. T4), *order of trials* (random-sequential vs. sequential-random) and *stimulation group* (10 Hz vs. 20 Hz vs. sham) weakens a confounding influence of the order of trials. Neither the main effect of *order of trials* (*F*_(1, 27)_ = 0.97, *p* = 0.33) nor the *time × order of trials × stimulation group* interaction (*F*_(6,81)_ = 1.98, *p* = 0.10) were found to be significant.

**Figure 4:**
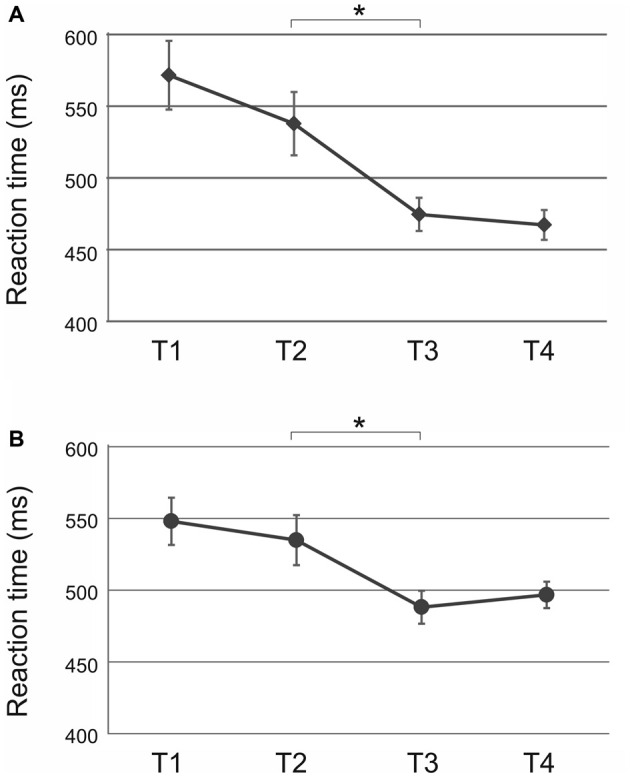
**Random condition: significant main effect of *time* following (A) tACS and (B) tDCS.** Reaction times were pooled across tACS and tDCS groups, respectively. Reaction times were significantly faster during retrieval (T3) as compared to the end of acquisition (T2). Shown are mean values. Error bars indicate the SEM. Asterisks indicate *p* < 0.05.

ANOVA for the random condition was performed analogous to the sequential condition with factors *time* (T1 vs. T2 vs. T3 vs. T4) and *tACS* (10 Hz vs. 20 Hz vs. sham). The analysis revealed a significant main effect of *time* (*F*_(3,81)_ = 8.91, *p* < 0.001; Figure 4A) but not of *tACS* (*F*_(2,27)_ = 0.58, *p* = 0.57) or a *time × tACS* interaction (*F*_(6,81)_ = 0.46, *p* = 0.77). Reaction times did not change significantly from baseline to end of acquisition (T1 vs. T2: *t*_(33)_ = 1.60, *p* = 0.24) but were faster during retrieval (T2 vs. T3: *t*_(33)_ = 3.30, *p* = 0.01) following each stimulation. No further improvement occurred during reacquisition (T3 vs. T4: *t*_(33)_ = 0.60, *p* = 0.55). Since the *time × tACS* interaction was not significant, further analyses for the random condition were not performed.

### Experiment 2 – tDCS

#### Does tDCS Yield Comparable Effects on SRTT Retrieval and Reacquisition?

ANOVA for the sequential condition revealed a significant main effect of *time* (*F*_(3,69)_ = 13.25, *p* < 0.001) but not of *tDCS* (*F*_(1,23)_ = 0.04, *p* = 0.85) or *time × tDCS* interaction (*F*_(3,69)_ = 0.83, *p* = 0.45) suggesting that reaction time improvement occurred independent of tDCS polarity. Reaction times improved from baseline to end of acquisition (T1 vs. T2: *t*_(27)_ = 1.74, *p* = 0.09 trend; Figure 2B). Reaction times further improved following tDCS (T2 vs. T3: *t*_(27)_ = 3.67, *p* = 0.01; Figure 3C). No significant improvement was observed from retrieval to reacquisition (T3 vs. T4: *t*_(26)_ = 0.67, *p* = 0.51; Figure 3D).

ANOVA for the random condition revealed a main effect of *time* (*F*_(3,66)_ = 6.51, *p* < 0.01; Figure 4B) but not of *tDCS* (*F*_(1,22)_ = 0.23, *p* = 0.63) or a *time × tDCS* interaction (*F*_(3,66)_ = 0.82, *p* = 0.49). Reaction times improved from end of acquisition to retrieval independent of tDCS polarity (T2 vs. T3: *t*_(26)_ = 3.01, *p* = 0.02). Since the *time × tDCS* interaction was not shown to be significant, analysis for the random condition was completed at this point.

### Experiments 1 and 2

Experiment 1 investigated frequency-specific effects of tACS, while Experiment 2 contrasted the polarity-specific tDCS effects on subsequent motor sequence retrieval and reacquisition. To draw a link between both experiments, ANOVA was calculated for the sequential condition with within-subject factor *time* (T1 vs. T2 vs. T3 vs. T4) and between-subject factor *stimulation group* (20 Hz tACS vs. anodal tDCS vs. cathodal tDCS). The ANOVA revealed a significant main effect of *time* (*F*_(3,99)_ = 24.97, *p* < 0.001) indicating that all three stimulation groups showed faster reaction times at the end of acquisition as compared to baseline (T1 vs. T2: *t*_(38)_ = 2.42, *p* = 0.04) and during retrieval as compared to the end of acquisition (T2 vs. T3: *t*_(38)_ = 5.07, *p* < 0.001). The significant main effect of *stimulation group* (*F*_(2,33)_ = 5.55, *p* = 0.01) showed that reaction times of the 20 Hz group were faster than those of the tDCS groups (anodal vs. 20 Hz *t*_(25)_ = −2.56, *p* = 0.03; cathodal vs. 20 Hz *t*_(25)_ = −2.64, *p* = 0.03). The *time × stimulation group* interaction (*F*_(6,99)_ = 0.58, *p* = 0.70) was not significant suggesting that 20 Hz tACS and tDCS yielded comparable effects on reaction times.

## Discussion

The present study investigates the effect of motor-cortical tACS and tDCS during early motor consolidation on subsequent sequence retrieval and reacquisition. Ten Hz, 20 Hz or sham tACS and anodal or cathodal tDCS were applied over left M1 immediately after SRTT acquisition with the right hand. Sequence retrieval (T3) was characterized by faster reaction times following 20 Hz tACS as compared to 10 Hz and sham tACS. Neither 10 Hz nor 20 Hz tACS yielded further reaction time improvement during reacquisition possibly indicating a ceiling effect. Following sham tACS only, reaction times improved during subsequent reacquisition (T4) suggesting that 20 Hz tACS is suitable to facilitate reaction times with less training.

Independent of polarity, tDCS yielded faster reaction times during retrieval (T3). No significant differences between tDCS and 20 Hz tACS effects on retrieval were found.

In both experiments, reaction times in the random condition were faster during retrieval (T3) as compared to end of acquisition (T2) suggesting a non-specific effect of stimulation and/or training.

### Beneficial Motor-Cortical Beta Band tACS Immediately After Motor Acquisition

The present main finding is indicated by facilitation of SRTT retrieval following 20 Hz tACS compared to 10 Hz and sham tACS. This finding adds to evidence from Antal et al. (2008) who showed a facilitation of reaction times when tACS was applied to M1 *during* SRTT acquisition. But importantly, that effect was frequency-specific for alpha tACS (10 Hz) only and not shown for beta tACS (15 and 30 Hz). In our previous study, both 10 and 20 Hz tACS applied *during* SRTT acquisition facilitated reaction times while 20 Hz tACS additionally favored motor stabilization as indicated by less interference to a random condition (Pollok et al., 2015). Our present and previous data may reveal a piece of evidence for the assumption that 20 Hz tACS applied to M1 is particularly effective on consolidation of a newly learned motor sequence. Taken together, one may conclude that motor sequence learning is differentially affected by tACS depending on frequency, timing (during vs. after acquisition) and state (during performance vs. at rest). But, acknowledging inconsistencies between studies, it has to be considered that tACS efficacy has been shown to vary depending on the power of ongoing oscillatory activity (Thut et al., 2011; Neuling et al., 2013; Helfrich et al., 2014). This conclusion is corroborated by Kanai et al. (2008) who showed that occipital cortex stimulation was most effective with frequencies that are dominant in light and darkness i.e., at beta frequency in an illuminated environment while strongest effects of alpha frequency stimulation were found in darkness (Kanai et al., 2008). Following sham tACS, no significant improvement was observed from end of acquisition to sequence retrieval. But, reaction times significantly improved from retrieval to reacquisition. This was not observed after 10 and 20 Hz tACS. This result implies that improvement of reaction times after further training – as shown in the sham condition – can be achieved by 20 Hz tACS with less training.

Although we do not exactly know whether tACS has indeed directly affected ongoing M1 oscillations, the data suggest a differential effect of 10 and 20 Hz tACS. Studies investigating neurophysiological brain mechanisms underlying motor control support the notion of increased beta oscillations’ inhibitory nature within neuronal motor control networks (Brown, 2003; Schnitzler and Gross, 2005). Accordingly, 20 Hz tACS has been shown to slow down voluntary movement *during* a visual coordination task without a significant impact on reaction time (Pogosyan et al., 2009). On the other hand, it has been argued that beta oscillations might be relevant for the maintenance of the current motor state (Engel and Fries, 2010) supporting the notion that higher beta power might be detrimental to motor learning. Along this line, a recent study revealed evidence for the assumption that the amount of beta power suppression during acquisition of a motor sequence is positively correlated with improvement of reaction times in a sequential learning task (Pollok et al., 2014). Thus, changes of 20 Hz oscillations may represent reorganization of M1 (Boonstra et al., 2007), and application of tACS in the range of the beta frequency band during consolidation may facilitate reorganization.

### Altered Motor-Cortical Excitability Immediately After Motor Acquisition

In Experiment 2, we examined to what extent motor sequence retrieval can be modulated by tDCS. Following anodal and cathodal tDCS, reaction times improved from acquisition to retrieval. This result extends the finding by Nitsche et al. (2003c) who observed faster reaction times *during* tDCS. Interestingly enough, reaction times in the present study were not differentially affected by anodal and cathodal tDCS. In previous studies, SRTT acquisition was facilitated with anodal tDCS as compared to sham (Nitsche et al., 2003c; Stagg et al., 2011). But, Nitsche et al. (2003c) observed a non-significant trend of reduced reaction times with cathodal tDCS, too. The authors argued that cathodal tDCS might modestly reduce cortical excitability and thus, cortical noise in general possibly leading to a focusing of cortical activity onto neurons involved in the learning process. Tecchio et al. (2010) applied tDCS for 15 min after acquisition i.e., during consolidation and found a beneficial effect of anodal tDCS as compared to sham. Noteworthy, participants learned with the non-dominant left hand a nine-digit-task and were stimulated above the right M1 with the second electrode placed above the ipsilateral arm. Since the beneficial effect was not evident in the random condition – in contrast to the present data, Tecchio et al. (2010) concluded that increased cortical excitability during early consolidation strengthens synaptic transmission in training-related neuronal networks. Beyond a mere modulation of cortical excitability by neuronal de- or hyperpolarization, tDCS effects additionally yield changes of neurotransmitter levels (Stagg et al., 2009). Furthermore, evidence from an animal model suggests that after-effects specifically of cathodal tDCS seem to be related to the adenosine A1 receptor (Márquez-Ruiz et al., 2012). Thus, tDCS effects are not exclusively polarity-dependent in the classical view leaving currently the question unanswered under which circumstances anodal and cathodal tDCS yield converse or diverse after-effects.

The present data suggest faster reaction times also in the random condition. Since this effect occurred also during sham tACS, we here would argue that practice with the task and the apparatus, rather than active tDCS/tACS may have yielded this improvement.

Vice versa, reduced motor-cortical excitability induced with slow repetitive transcranial magnetic stimulation (rTMS) was associated with a disruption of early memory consolidation when applied immediately after training (Muellbacher et al., 2002; Robertson et al., 2005). These studies are in line with the assumption that M1 is relevant for motor learning as well as for early consolidation of the newly learned skill (Reis et al., 2008).

By showing that the reaction time improvement from end of acquisition to retrieval was comparable between 20 Hz tACS and tDCS, we here would argue that beta band tACS after-effects on motor sequence retrieval may be associated with altered motor-cortical excitability. Although we realize that this interpretation is highly speculative without neurophysiological measures, it is in line with the notion that with prolonged stimulation duration tACS is associated with altered motor-cortical excitability and/or neuroplastic reorganization (Antal and Paulus, 2012; Thut et al., 2011; Herrmann et al., 2013).

### Limitations

A major limitation of the present study is a lack of direct neurophysiological measures. Thus, we can only speculate about the mechanisms underlying the effects reported above. Nevertheless, the results suggest a facilitatory effect of 20 Hz tACS as well as tDCS. This accordance might imply that the effects following 20 Hz tACS might occur due to alterations of M1 excitability although this interpretation cannot be directly justified by the present data. We acknowledge that a baseline difference in reaction times was evident between 20 Hz tACS and tDCS which might partly have driven the difference during retrieval, too. On the other hand, no differential effects of 20 Hz tACS and tDCS were shown revealing a piece of evidence that altered excitability may have contributed to superior retrieval following 20 Hz tACS.

Another limitation of the present study is that motor sequence retrieval was measured 10 min after the end of acquisition only – allowing a conclusion about immediate stimulation but not persisting effects on early motor consolidation.

Furthermore, we cannot completely rule out that stimulation may have affected the premotor or prefrontal cortex both directly induced by stimulation with relatively large 7 × 5 cm^2^ electrodes and indirectly induced by network effects in functionally connected areas of the left M1. The premotor cortex is part of the network underlying motor sequence learning (Hardwick et al., 2013). Muellbacher et al. (2002) showed that procedural (or implicit) learning and its early consolidation are rather mediated by M1 and not premotor cortex. As opposed to M1 which is particularly relevant during initial acquisition and early consolidation of motor sequences (Halsband and Lange, 2006), the premotor cortex is assumed to be rather involved in late consolidation in particular during sleep (Nitsche et al., 2010) weakening a direct premotor contribution to the observed effects. We, furthermore, cannot exclude the possibility of right prefrontal cortex co-stimulation due to the return electrode’s position above the right orbita. The prefrontal cortex is also part of the network involved in SRTT learning but might rather contribute to explicit learning (Halsband and Lange, 2006; Kantak et al., 2012). Moreover, Nitsche et al. (2003c) and Kantak et al. (2012) previously showed a benefit of anodal M1 tDCS on SRTT acquisition while no significant effect was observed with premotor and prefrontal cortex tDCS (Nitsche et al., 2003c), or even an adverse effect was found with premotor cortex tDCS (Kantak et al., 2012). Thus, we here would argue that tACS and tDCS effects are likely due to stimulation of M1.

Finally, we acknowledge that sham tACS blinding was not optimal since the rate of identification (10/12 participants) was above the level of guessing. Thus, we cannot definitely rule out the possibility that knowledge regarding the stimulation frequency might have contributed to the results reported here. Nevertheless, the lack of blinding cannot explain the distinct effects of 10 Hz tACS rendering a significant confound less likely.

## Conclusion

20 Hz tACS applied to M1 immediately after SRTT acquisition facilitates reaction times during sequence retrieval shortly after initial training on a motor sequence as compared to 10 Hz and sham tACS. Although we did not determine the effect of tACS and tDCS on neurophysiological measures, this behavioral stimulation effect should be seen as further evidence supporting the hypothesis that altered motor-cortical beta oscillations might represent functional reorganization subserving motor sequence learning.

## Author Contributions

VK and BP designed the experiment. AM and LD performed data acquisition and analysis. All authors contributed to the interpretation of results. VK drafted the manuscript. BP revised the manuscript for intellectual content. All authors approved the final version of the manuscript.

## Conflict of Interest Statement

The authors declare that the research was conducted in the absence of any commercial or financial relationships that could be construed as a potential conflict of interest.

## Abbreviations

EHI: Edinburgh Handedness Inventory
FDI: first dorsal interosseous muscle
M1: primary motor cortex
MEG: magnetoencephalography
rTMS: repetitive transcranial magnetic stimulation
SRTT: serial reaction time task
tACS: transcranial alternating current stimulation
tDCS: transcranial direct current stimulation
TMS: transcranial magnetic stimulation.
